# Plasmonic photoluminescence for recovering native chemical information from surface-enhanced Raman scattering

**DOI:** 10.1038/ncomms14891

**Published:** 2017-03-28

**Authors:** Kai-Qiang Lin, Jun Yi, Jin-Hui Zhong, Shu Hu, Bi-Ju Liu, Jun-Yang Liu, Cheng Zong, Zhi-Chao Lei, Xiang Wang, Javier Aizpurua, Rubén Esteban, Bin Ren

**Affiliations:** 1 Collaborative Innovation Center of Chemistry for Energy Materials, State Key Laboratory of Physical Chemistry of Solid Surfaces, and The MOE Key Laboratory of Spectrochemical Analysis and Instrumentation, Department of Chemistry, College of Chemistry and Chemical Engineering, Xiamen University, 361005 Xiamen, China; 2 Donostia International Physics Center (DIPC) and Material Physics Center (CSIC-UPV/EHU), Paseo Manuel de Lardizabal 4, 20018 Donostia-San Sebastián, Spain; 3 IKERBASQUE, Basque Foundation for Science, Maria Diaz de Haro 3, 48013 Bilbao, Spain

## Abstract

Surface-enhanced Raman scattering (SERS) spectroscopy has attracted tremendous interests as a highly sensitive label-free tool. The local field produced by the excitation of localized surface plasmon resonances (LSPRs) dominates the overall enhancement of SERS. Such an electromagnetic enhancement is unfortunately accompanied by a strong modification in the relative intensity of the original Raman spectra, which highly distorts spectral features providing chemical information. Here we propose a robust method to retrieve the fingerprint of intrinsic chemical information from the SERS spectra. The method is established based on the finding that the SERS background originates from the LSPR-modulated photoluminescence, which contains the local field information shared also by SERS. We validate this concept of retrieval of intrinsic fingerprint information in well controlled single metallic nanoantennas of varying aspect ratios. We further demonstrate its unambiguity and generality in more complicated systems of tip-enhanced Raman spectroscopy (TERS) and SERS of silver nanoaggregates.

## Abstract (web summary)

Localized surface plasmon resonances induce strong modifications of surface-enhanced Raman spectra. Here the authors propose a robust method to retrieve the fingerprint of intrinsic chemical information by using the photoluminescence of the metallic structures to correct the spectra.

Localized surface plasmon resonances (LSPRs)^1^ in metallic nanostructures can confine incident light into a nanoscale volume characterized by strongly enhanced optical fields. These hot-spots can dramatically enhance the Raman scattering^2^,^3^,^4^,^5^, fluorescence^6^,^7^, infrared absorption^8^, or nonlinear optical signal^9^, leading to a family of plasmon-enhanced spectroscopies. These techniques have become powerful tools for identification and characterization of molecular species related to chemistry, energy, material sciences, and physics and for analysis of bio-related systems^10^,^11^,^12^, owing to their extraordinary sensitivity reaching single molecule level^4^,^5^,^13^,^14^,^15^. Notably, the relative intensity of Raman peaks in both surface-enhanced Raman scattering (SERS) and tip-enhanced Raman scattering (TERS) has been widely used to investigate the surface configuration of adsorbed molecules^13^,^16^,^17^, charge transfer mechanism^18^,^19^, local temperature^20^,^21^, vibrational pumping^20^,^22^, plasmon-mediated photocatalysis^23^,^24^ and local chemical properties^25^. However, it was frequently observed that the relative intensity of different Raman peaks may vary from spot to spot even on the same surface, which is unexpected from the chemical interaction point of view because the chemical properties should remain similar^18^,^21^,^26^. Importantly, LSPRs do not only provide a large electromagnetic enhancement but also induce strong modifications of the relative strength of the peaks in the SERS spectra. The latter effect can be seen as a plasmonic spectral shaping effect (PSSE)^21^. The distorted spectral features as a result of the PSSE can severely undermine the interpretation of the experimental data when extracting chemical information like molecular orientation^13^,^17^, the charge transfer mechanism in plasmon-mediated photocatalysis^23^,^24^, local chemical properties^25^ and so forth. It is thus desirable to reach a deeper understanding of the PSSE and to develop a method to recover the native chemical information.

The plasmon-induced PSSE was studied by Itoh *et al*., who successfully demonstrated a correlation between the relative strength of the SERS peaks of the molecules and the scattering of the SERS substrate^21^,^27^. The first reported method to directly correct the PSSE was successfully demonstrated by Buchanan *et al*. using what they identified as the incompletely quenched surface-enhanced fluorescence (SEF) signal of dye molecules on SERS substrates^28^. An advantage of this method is that the SEF is directly affected by the near field response^28^, as in the case for the PSSE. However, this method is not applicable to more general systems in the absence of dye molecules or with strongly quenched fluorescence. Due to these limitations, no robust and systematic procedure has been broadly adopted by the SERS community to treat the PSSE with generality. As a result, information from ‘untreated' Raman spectra is still constantly used as the basis to reach incorrect conclusions regarding selection rules in vibrational spectroscopy, a situation detrimental for further developments of SERS, and even of other fields related to plasmon-enhanced chemical reactivity or two-dimensional materials spectroscopy. Thus, it is important to develop a general method to correct for the PSSE that is robust and universally adoptable.

The photoluminescence (PL) of metal nanostructures has been connected to the behavior of the near fields inside the nanoparticle^29^,^30^,^31^,^32^,^33^ and has been considered to contribute to the broad ‘continuum' background in typical SERS spectra^31^,^34^,^35^. This background is commonly neglected or just directly subtracted to obtain a clean SERS spectrum^4^,^5^,^36^, but its origin as PL means that it should contain similar near field information as the PSSE. Therefore, the SERS background may potentially be used to correct the PSSE and to recover the intrinsic chemical information contained in the relative strength of the Raman peaks.

Here we use single-gold nanorods as nanoantennas, which are resonant at different wavelengths but show the same chemical properties, to demonstrate such concept of correction. We carefully correlate their morphology, LSPR scattering, PL, SERS background and relative intensity of the SERS peaks (Fig. 1). We identify the contribution from the material response and the plasmonic enhancement to the PL through measuring the scattering and the PL response of the individual nanorods. We further quantitatively connect the PL and SERS background by measuring the PL and SERS of single nanorods before and after the adsorption of molecules, respectively. Based on these results, we propose a robust method to correct the PSSE that consists in normalizing the SERS by the SERS background. We successfully apply this approach to resolve and understand the PSSE signal in nanorods system, and corroborate the results by analytical models and numerical simulations. The generality of the proposed method is further supported by the results from more complex plasmonic systems including gap-mode TERS configurations and silver nanoaggregates.

## Results

### Correlating the PL with the LSPR response

**Figure 2:**
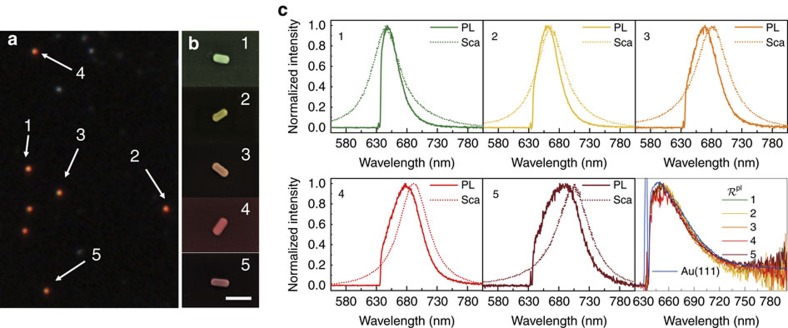
Correlation of the PL and LSPR scattering for single gold nanorods. (**a**) Dark-field scattering image of the sample, where the five nanorods with LSPRs at different wavelengths are marked by arrows. (**b**) SEM images of the nanorods indicated in **a** in pseudo color. Scale bar, 200 nm. (**c**) 1–5: normalized PL spectra (solid lines) excited at 633 nm and elastic scattering spectra (dotted lines) excited with a white light source, for the five nanorods selected in **a**,**b**. Bottom right panel: Normalized ratio of the PL spectra to the scattering spectra, 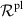, of these five nanorods (labeled 1–5) and the PL spectra of a gold(111) single-crystal surface normalized by the value at 648nm (blue line).

We first connect the morphology of the individual nanorods and their single-nanoparticle optical response (scattering and PL) using a quartz substrate with microfabricated markers (Supplementary Fig. 1) for co-localization between the dark-field microscope and the scanning electron microscope (SEM). We choose a first set of five gold nanorods with different aspect ratios for the following experiments, which are marked in the dark-field image (Fig. 2a) and characterized by SEM (Fig. 2b). The PL (solid lines in Fig. 2c) and scattering (dotted lines in Fig. 2c) spectra of the five nanorods were excited with a 633 nm continuous wave (CW) laser and a white source, respectively, and were collected through the same objective using a home-built combined dark-field microscope and Raman microscope system (See schematic diagram of the system in Supplementary Fig. 2). To increase the signal-to-noise ratio, the polarization of both the laser and white light were kept parallel to the longitudinal axis of the nanorods in all measurements. We observe from Fig. 2c that, similarly to what has been reported in the literature, the PL spectra of the five gold nanorods show a prominent peak (in the 633 nm–720 nm range) that roughly follows the LSPR scattering spectra^37^. However, the PL peaks are blue-shifted with respect to the scattering peaks^38^,^39^ and the shifts depend on the resonant energy, becoming larger for the nanorods of larger aspect ratio that resonate at longer wavelengths.

The physical origin of PL of gold nanostructures is still under debate^30^,^31^,^34^,^37^,^39^,^40^, but we consider a simple model that explains our PL measurements without invoking the detailed mechanism by which the energy is dissipated. Thus, by PL we mean here the emission at a smaller energy of the incident laser. We treat the PL as an incoherent process (phases are ignored) initiated by the incoming light of amplitude *E*_0_ and angular frequency *ω*_exc_, polarized along the nanorod axis. The excitation efficiency is proportional to the local field intensity 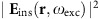 at each position **r**, where 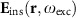 is the field induced inside the nanostructures. During the PL processes, optical dipoles (which can also be seen as currents oscillating at optical frequencies) are excited at the emission angular frequency *ω*_em_ (energy loss 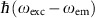) with a probability 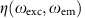, and emit at a rate 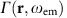. We thus obtain a local PL signal proportional to 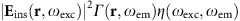. Using reciprocity^41^,^42^ to relate the emission rate from a dipole to the local field enhancement at the same angular frequency (see Supplementary Notes 1 and 2, and Supplementary Figs 4 and 5), we write the emission rate as 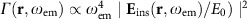, where 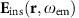 is the local field induced by a plane wave of angular frequency *ω*_em_ polarized along the axis of the nanorod (equivalent to 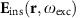 but for the emission frequency). The 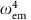 factor describes the frequency-dependence of the emission from a dipole of a fixed strength. After integrating over the volume 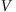 of the nanoparticle, we obtain an expression, consistent with previous work^32^,^33^:


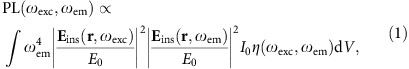


where 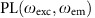 is the photoluminescence emission at *ω*_em_ for excitation at *ω*_exc_ and 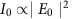 is the incident intensity.

The dependence of the PL on the local fields will be a key below when discussing the method to correct the PSSE on the SERS signal, but it makes the direct verification of equation (1) more difficult, because 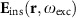 is not easily accessible in experiments. Fortunately, for simple systems where a single resonance dominates the response and the radiative damping is low^43^, the scattering *S*(*ω*_em_) and the square of the local fields 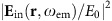 exhibit a similar energy dependency. Under this assumption, the PL can be expected to depend on the emission frequency approximately as:


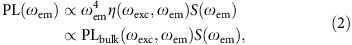


where 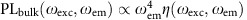 captures the bulk properties of the material. As a consequence, we should obtain the same *ω*_em_-dependence of the ratio between photoluminescence and scattering 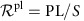 when comparing different nanostructures. This expectation is confirmed experimentally, as shown in the results at the bottom right of Fig. 2c, where we show an almost identical 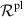 for the five different nanorods under study. Furthermore, we also measured the PL of an atomically smooth gold(111) single-crystal surface (PL_bulk_, blue line in bottom right of Fig. 2c), as essentially corresponding to the bulk gold PL response. Indeed, the spectral shape for this measurement is almost identical to the 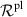 ratio from the nanorods, in agreement with the description of the PL obtained in equation (2). Nonetheless, we emphasize that, in general, the PL should depend on the near field as given by equation (1), and that the use of *S*(*ω*_em_) in equation (2) and Fig. 2c would only be valid for particular cases. The derivation of equations (1) and (2), and the conditions where the different approximations are valid are discussed in more detail in Supplementary Notes 2 and 4, and Supplementary Figs 5 and 8 (particularly how some of the relatively small, higher order corrections partially cancel, giving an almost perfect agreement in the bottom right panel of Fig. 2c).

### Quantitative relationship between PL and the SERS background

**Figure 3:**
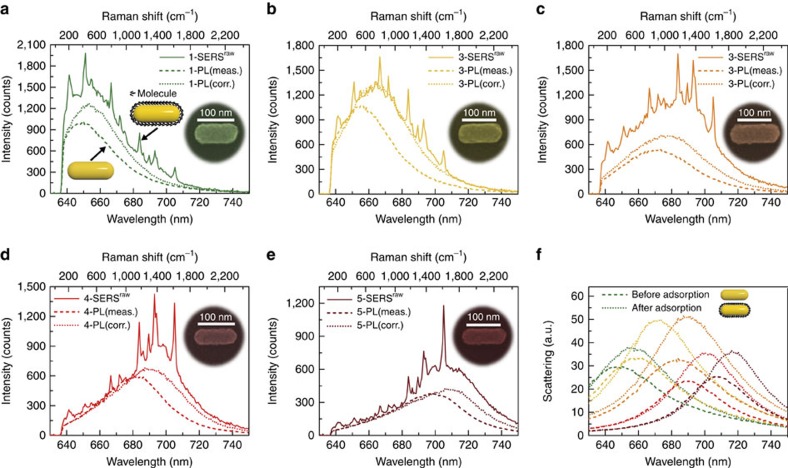
Quantitative correlation of the SERS background and PL for single gold nanorods. (**a**–**e**) Measured PL signal, PL(meas.) (dashed lines), of five gold nanorods with different aspect ratio in the absence of molecules and the corresponding SERS^raw^ spectra (which includes a spectrally broad background) after adsorption of the MGTIC molecules (solid lines). All measurements were performed under 633 nm laser illumination. The dotted lines, PL(corr.), correspond to the PL after applying the correction described by equation (3), which accounts for the influence of the molecules on the plasmonic resonance. The insets are the SEM images of the five nanorods in pseudo color, which show an increased aspect ratio from **a**,**e**. Scale bar, 100 nm. (**f**) Scattering spectra of the five gold nanorods measured before (dashed lines) and after (dotted lines) adsorption of the MGITC molecule.

Since PL is due to the intrinsic response of the nanorods, we expect it to appear as a background in the SERS spectra^31^,^34^,^35^. To assess this connection quantitatively, we successively measure the PL (dashed lines in Fig. 3a–e) and SERS^raw^ signal (solid lines in Fig. 3a–e) of another set of five nanorods of different aspect ratio (SEM images in the insets of Fig. 3) before and after molecule adsorption, respectively. We introduce the superscript ‘raw' to specify that we are discussing the Raman signal before subtracting the broad background. The Raman signal of non-resonant molecules adsorbed on a single Au nanorod is too weak to perform a reliable analysis. Therefore, we chose the functionalized malachite green isothiocyanate (MGITC) molecules (see Supplementary Fig. 3 for the molecular structure) as the target molecule, which has an absorption peak at about 630 nm, in resonance with the 633 nm excitation laser and thus ensuring a sufficiently high signal-to-noise ratio of single-nanoparticle SERS spectra. To avoid a strong fluorescence background from molecules adsorbed on the quartz substrate, the substrate was first modified by trichloro(1H, 1H, 2H, 2H-perfluorooctyl)silane (PFS) to impede the adsorption of the MGITC (see Methods for detailed experimental procedures). The use of the same molecule, nanorods synthesized in the same vessel and the same excitation laser strongly attenuate the possible contribution of different chemical effects to the SERS spectra.

Furthermore, the PL and SERS were measured on the same five nanorods, with the same optical set-up and under identical conditions (except for whether the molecules are present). The PL and SERS spectra in Fig. 3 can thus be directly and quantitatively compared, including the absolute strength. Crucially, we find that the SERS background, Bg, in presence of molecules closely resembles the PL spectrum before the molecules are deposited, in both spectral profile and strength. This similarity is observed for all the five nanorods with resonances at different frequencies, which clearly demonstrates that PL is the main contributor to the SERS background in the present system (this finding is further stressed by the more detailed analysis below); other possible contributions, such as the SEF of a residual amount of MGITC on the quartz substrate, are comparatively weak.

It can also be noted that there is a small red shift in the position of the SERS background with respect to that of the PL peak for all the five gold nanorods in Fig. 3a–e. We attribute the difference to the red shift of the LSPR induced by an increase of the refractive index outside the nanorod surface after adsorption of MGITC molecules. According to equation (2), the PL from simple systems is approximately proportional to the LSPR scattering. Therefore, to account for the effect of molecular adsorption on PL, we analyse the scattering spectra of the five nanorods before (dashed lines in Fig. 3f) and after (dotted lines in Fig. 3f) adsorption. After adsorption, the scattering spectra show significant red shift in wavelength and an increase in intensity, compared with that of bare gold nanorods, which agrees well with simulations of the optical response based on the finite-element method (Supplementary Figs 4 and 6). With the scattering spectra *S* and the measured PL, PL(meas.) on hand, the corrected PL spectra, PL(corr.) can be written from equation (2) as


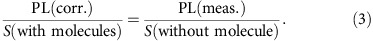


Significantly, the corrected PL spectra (dotted lines in Fig. 3a–e) of all five nanorods show a significantly better match with the SERS backgrounds both in the peak position and the absolute intensity. This agreement further supports the validity of equation (2), and thus substantiates that the PL to scattering ratio, 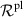, corresponds in this case to the intrinsic response of bulk gold and is independent of the LSPR of nanorods.

### Correcting the relative intensity of the SERS peaks using the PL

**Figure 4:**
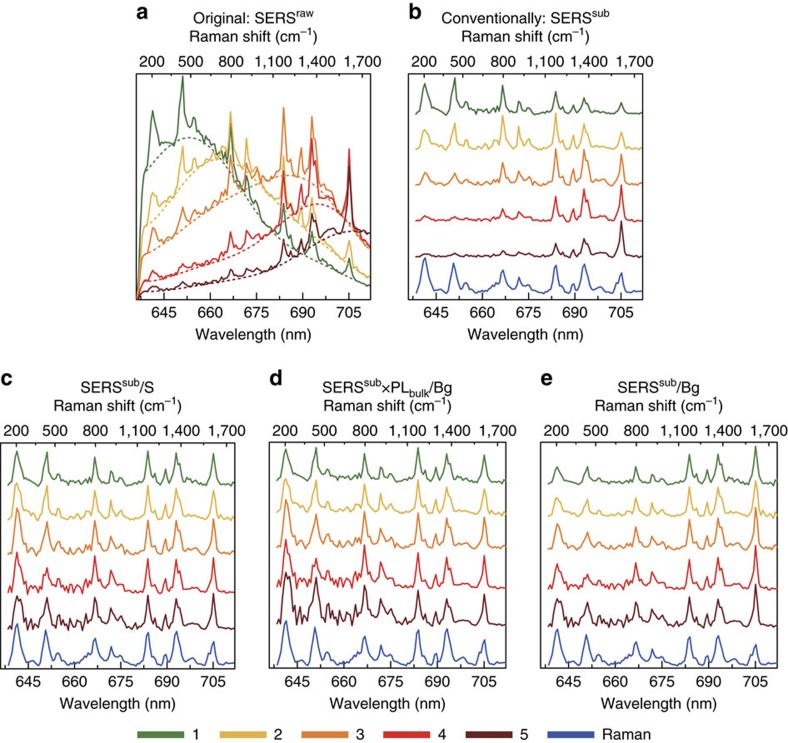
Suppressing the PSSE to retrieve the intrinsic relative intensity of the Raman speaks. (**a**) SERS^raw^ spectra from Fig. 3a–e, corresponding to five nanorods of different aspect ratio before background subtraction, plotted here in a single graph for simpler comparison. (**b**) SERS^sub^ spectra obtained after subtracting the fitted SERS background Bg to the spectra in **a**. (**c**–**e**) Spectra obtained by normalizing each of the five SERS^sub^ spectra in **b**. In **c** a normalization by the corresponding elastic scattering spectrum of each nanorod (dotted lines in Fig. 3f) is used to obtain SERS^sub^*/*S. (**d**) SERS^sub^* × *PL_bulk_*/*Bg, obtained by dividing each background-subtracted SERS spectrum by the corresponding fitted SERS background and then multiplying each spectrum by the PL_bulk_ signal in the bottom right panel in Fig. 2c. (**e**) SERS^sub^*/*Bg ratio, obtained after normalization by the SERS background. For comparison, the normal Raman spectrum of MGITC on a gold (111) single-crystal surface is shown at the bottom (blue line) of **b**–**e**. All the spectra in **c**–**e**, have been further normalized by the corresponding SERS^sub^ peak at 683.8 nm (corresponding to 1,173.3 cm^−1^ in Raman shift).

The understanding of the PL and its connection with the SERS background is used next to justify the main result of this paper, that is, how to suppress the PSSE from SERS measurements to retrieve intrinsic molecular fingerprint information. With this aim, we first plot in a single panel (Fig. 4a) all the SERS^raw^ spectra shown separately in Fig. 3, corresponding to different aspect ratios of the nanorods and thus to different LSPR wavelengths. The relative strength of the peaks varies strongly from nanorod to nanorod. This point is further emphasized in Fig. 4b, where the background of the SERS spectra is first fitted and then subtracted (see Supplementary Note 5 for the detailed procedure) to obtain clean SERS spectra. In the following, we refer to the signal after background subtraction (SERS^sub^=SERS^raw^–Bg), which allows to focus on the narrow Raman peaks. For Nanorod 1, the LSPR peak is at around 648.2 nm and the Raman peak corresponding to a 438.7 cm^−1^ Raman shift (emission at 651.1 nm) has the highest intensity, with the Raman peak for 1,614.5 cm^−1^ (705.1 nm) exhibiting a much lower intensity. In contrast, for Nanorod 5 supporting a LSPR at 708.9 nm, the 1,614.5 cm^−1^ (705.1 nm) Raman peak becomes the strongest and the Raman peaks for 205.2 cm^−1^ (641.3 nm) and 438.7 cm^−1^ (651.1 nm) are significantly weaker. As previously introduced, we discard changes on the chemical interaction between molecules and nanorods as an explanation of such a drastic change in the relative SERS intensity, because all the nanorods were synthesized in the same flask and thus chemical interactions should be the same.

In contrast, the PSSE provides a robust understanding of the observed changes because, for all the five nanorods, the Raman peaks are more clearly enhanced when their corresponding emission energy is close to the peak position of the LSPR spectra. It is apparent from Fig. 4a,b that the PSSE can be very significant, and it is crucial to consider it for a reliable interpretation of the measurements. For example, it has been pointed out by Itoh *et al*.^21^ that the temperature of a molecule could be erroneously estimated if the relative intensity of the anti-Stokes and Stokes lines are directly compared without considering the plasmonic response, up to the point of inferring a temperature that exceeds the decomposition temperature of the target molecule.

We argue next that the spectral behavior of the PL (and thus of the SERS background) and of the PSSE should resemble each other because both depend on the near fields in a similar manner, which facilitates disentangling the chemical information from the electromagnetic contribution to SERS. The electromagnetic Raman enhancement is proportional to both the local intensity enhancement at the illumination frequency (excitation) and the radiation enhancement (emission)^44^. As in the discussion of the PL, due to reciprocity, the latter is typically proportional to the local intensity enhancement at the emission frequency. The intensity of the SERS peaks from the different vibrational modes of the molecule can then be expressed as


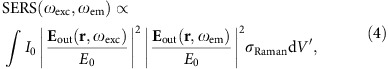


where 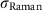 is the Raman cross section of the molecule containing the chemical information and 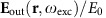 and 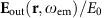 are the local field enhancement factor at the excitation and emission frequencies, respectively, evaluated at the position of the molecules, that is, outside the nanoparticle. The PSSE in our experiment would be a consequence of the dependence of the local near fields on *ω*_em_ in equation (4).

The near fields are not easily accessible to experiments^45^,^46^, which makes the direct evaluation of equation (4) to extract 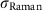 a considerable challenge. A first alternative is to follow a similar approach as used in equation (2), and to consider the scattering *S*(*ω*_em_) at the emission frequency to be roughly proportional to the square of corresponding near fields (see Supplementary Fig. 7b). We can simplify equation (4) to


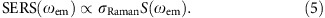


Under adequate conditions, this simple relationship can be sufficient to reveal the relative value of 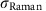 for the different Raman peaks, as experimentally observed by Itoh *et al*.^21^,^27^. We experimentally corroborate that we can effectively suppress the PSSE for the nanorods by dividing the background-subtracted SERS^sub^ spectra in Fig. 4b by the corresponding scattering spectra (dotted lines in Fig. 3f). The resulting 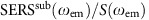 spectra (Fig. 4c) is indeed very similar for all the five nanorods of different aspect ratio, in clear contrast with the large differences before the correction (Fig. 4b). Moreover, the five normalized spectra are almost identical to the Raman signal of the same molecules adsorbed on a gold(111) single-crystal surface (shown in the figure by the bottom blue line), further evidence that the normalized results indeed correspond to 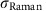.

For well-defined dipolar modes as those presented here, the normalization procedure suggested by equation (5) appears as reliable and robust. However, we do not expect equation (5) to be valid with generality, because the connection between the near fields and the scattering can become very complex in a general nanoantenna configuration. These more challenging scenarios can include, for example, structures that support several plasmonic modes at the relevant frequencies, or systems whose far field is dominated by a non-resonant background unrelated to the modes dominating the near field. Furthermore, scattering spectra can be difficult to obtain in typical SERS and TERS experiments without integrated dark-field microscopy in the Raman system.

To circumvent these difficulties, it is convenient to compare the equations that show explicitly the dependence of the PL signal and the Raman enhancement on the near fields (equation (1) and equation (4)). Identifying the SERS background, Bg, as the PL (Fig. 3), using 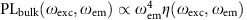, and assuming that the integrals over the local field intensity inside (equation (1)) or outside (equation (4)) the metal nanostructure exhibit a similar *ω*_em_-dependence (see Supplementary Notes 3,4 and Supplementary Fig. 9 for a more detailed discussion of the derivation and approximations involved), we obtain


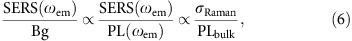


and the intrinsic Raman signature of the molecule can thus be written as


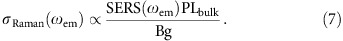


We evaluate equation (7) by dividing the background-subtracted SERS^sub^ spectrum of each nanorod by the corresponding SERS background and then multiplying by the previously obtained PL_bulk_ (PL measured for a gold(111) single crystal, Fig. 2c), SERS^sub^* × *PL_bulk_/Bg. The detailed data processing procedure, and the comparison between the fitted background and the PL of the same nanorods before molecule adsorption are provided in the Supplementary Note 5 and Supplementary Fig. 10. The obtained corrected Raman spectra are again almost identical for all the five nanorods (Fig. 4d), and very similar to the spectra measured for the molecules on a gold(111) surface (blue line). Thus, the correction scheme given by equation (7) behaves as intended for retrieving the intrinsic information from the molecule.

It is interesting to compare our approach with that reported by Buchanan *et al*.^28^, who directly normalize the SERS spectra with the SERS background. We emphasize that they identified their broad background in the SERS experiments not as PL from the metal but as SEF from the incompletely quenched dye molecules. The SEF would thus play a similar role in their work as the PL in our results. However, if we use the background to directly correct the spectra (Fig. 4e), that is, without the PL_bulk_ factor, we can identify a noticeable difference in the relative intensity for the two peaks at 641.3 nm (Raman shift 205 cm^−1^) and 705.1 nm (1,614 cm^−1^; Fig. 4e), when comparing the results for the nanorods with those for the gold single-crystal surface. Thus, the correction of the PSSE to recover the intrinsic Raman spectra is more robust and systematic when we use the full equation (7), including the intrinsic PL of the gold.

### Generalization to TERS and more complicated SERS systems

Equation (7) is obtained by relating two magnitudes, the PL and the SERS enhancement, both of which directly depend on the near fields. As a consequence, calculating SERS^sub^* × *PL_bulk_/Bg should also allow to recover the intrinsic chemical information for rather general systems. To evaluate the validity of our understanding and the generality of the correction method, we further applied it to tip-substrate (gap-mode TERS) and silver nanoaggregate systems.

**Figure 5:**
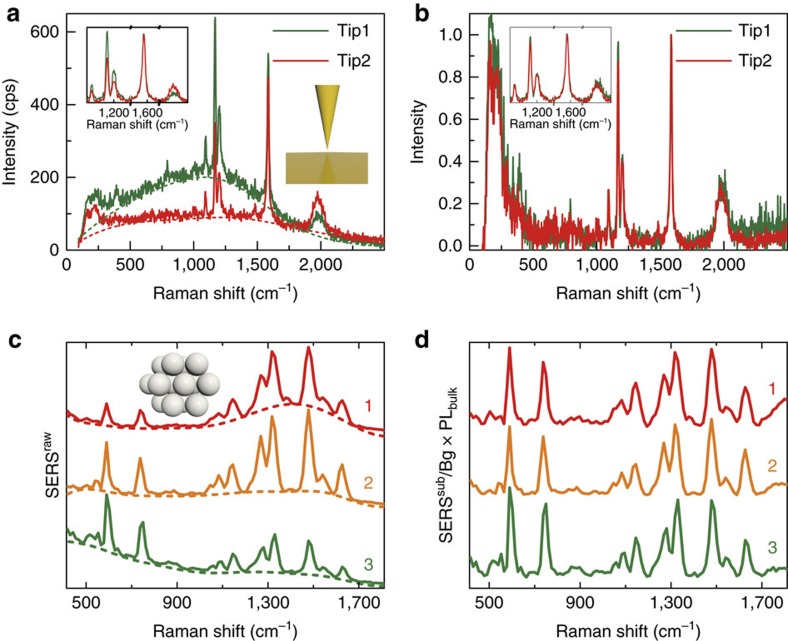
TERS signal and SERS spectra of silver nanoaggregates before and after correction. (**a**) TERS^raw^ spectra for a gold tip near a flat gold surface, the latter covered by 4-chlorophenyl isocyanide molecules (solid lines), and corresponding background Bg (dashed lines). The two sets of spectra are obtained using two different tips. The inset shows the background-subtracted TERS^sub^ spectra in the region 1,000–2,100 cm^−1^, which were further normalized to the Raman peak at 1,586 cm^−1^. (**b**) Corrected TERS spectra obtained from the spectra in **a** by subtracting the background Bg and processing the resulting TERS^sub^ according to TERS^sub^* × *PL_bulk_/Bg, which were further normalized with the Raman peak at 1,586 cm^−1^. The inset shows the enlarged corrected TERS spectra in the region 1,000–2,100 cm^−1^. (**c**) Three typical SERS^raw^ spectra of Rhodamine 6G molecules adsorbed on different silver nanoaggregates (solid lines), and corresponding background (dashed lines). (**d**) Corrected SERS spectra obtained from the spectra in **c** by subtracting the background Bg and processing the resulting SERS^sub^ as SERS^sub^* × *PL_bulk_/Bg, which was further normalized to the Raman peak at 1,545 cm^−1^.

As shown in Fig. 5a, two TERS spectra were obtained with two different gold tips on the same one-atom-thick palladium layer deposited on a gold(111) single-crystal surface adsorbed with 4-chlorophenyl isocyanide molecules. In this case, the illumination at 633 nm is off-resonant with the electronic transitions of the molecule. Both the background and the relative intensity of the main Raman peaks of the two TERS spectra are quite different. In the case of tip1, the intensity of the Raman peak at 1,168 cm^−1^ is slightly stronger than that at 1,586 cm^−1^ (after background subtraction, see inset). Inversely, for tip2 the intensity of the Raman peak at 1,168 cm^−1^ is significantly weaker than that at 1,586 cm^−1^. In TERS, the tip is not in contact with the molecule, and the interaction of the molecule with the substrate should be the same for the two measurements, so that we safely attribute the changes to differences on the plasmonic response. Following the correction method introduced in Fig. 4d, by dividing the background-subtracted TERS spectra by their own fitted backgrounds (Fig. 5a, dashed lines) and multiplying by the bulk gold PL (Fig. 2c, blue line), that is, TERS^sub^* × *PL_bulk_/Bg, we obtain almost the same spectrum for the two tips (Fig. 5b).

Last, we consider a canonical SERS substrate such as that composed of silver nanoaggregates made of chemical synthesized silver colloids. These complex plasmonic systems are widely used SERS substrates and allow to test the correction method for a different material showing complex couplings between the constituent nanoparticles. Three typical SERS^raw^ spectra of Rhodamine 6G molecules on the silver nanoaggregates excited at 633 nm (off-resonance) are shown in Fig. 5c, which again exhibit different relative intensity of the Raman peaks. By dividing the three background-subtracted SERS spectra by their own fitted background (Fig. 5c, dashed lines) and multiplying by the bulk silver PL, SERS^sub^ × PL_bulk_/Bg, we obtain three spectra (Fig. 5d) with remarkably similar relative intensity of the different Raman peaks, further validating the generality of the proposed method for retrieving intrinsic fingerprint information.

## Discussion

In conclusion, we have proposed a general approach to correct the influence of the plasmon resonance dispersion on the relative intensity of the SERS peaks, known as the PSSE, to retrieve intrinsic information of the Raman signal of molecules. Our intrinsic fingerprint retrieval method exploits the PL-induced SERS background as an intrinsic spectral feature (also an internal standard) that contains the same near field information as the PSSE, and allows to recover molecular fingerprints that reflect the intrinsic chemical interaction of the molecule with the substrate. From a practical perspective, once the PL_bulk_ is known, which should only depend on the material and the illumination wavelength, our method can be applied to standard SERS or TERS experiments, without requiring any additional measurements. In particular, as our procedure does not rely on the scattering signal, it can be applied to situations where the far field signal is difficult to measure or is uncorrelated to the near fields, that is, to the SERS enhancement. After correcting the raw Raman signal with the method proposed here, the corrected spectra may provide a more reliable starting point to explore complex phenomena, related to the molecule orientation, to changes on the selection rules due to the interaction with the metallic particle, to vibrational pumping or other strong field effects, or to very large field gradients. This method can also serve as a powerful tool to clarify the origin of the photoluminescence, an important but yet controversial issue^47^.

The intrinsic fingerprint retrieval procedure seems particularly necessary for systems that are expected to show a strong PSSE, such as SERS substrates with narrow and well-defined LSPR, and SERS of single or few molecules that only probes a certain hot spot with narrow LSPR mode at a time. In contrast, for many molecules over highly disordered SERS substrates, the PSSE could smear out and the correction may become less relevant. We have successfully applied the method to resonant and non-resonant Raman molecules, different plasmonic materials and three typical systems: single nanorods (as a well-defined model system), TERS and nanoaggregates (of direct relevance to practical application). A general protocol to correct the PSSE as presented here could be very useful in analytical sciences, and opens a new avenue towards unambiguous retrieval of native chemical information from the relatively complex and distorted information of Raman peaks in SERS and TERS spectra.

## Methods

### Sample preparation

We synthesized the gold nanorods following a reported binary surfactant method^48^. The gold nanoseeds were synthesized first and a growth process was performed 30 min afterwards. The nanoseeds were synthesized by injecting freshly prepared sodium borohydride (NaBH_4_) (0.6 ml, 0.01 M) into the HAuCl_4_–hexadecyltrimethylammonium bromide (CTAB) mixture (HAuCl_4_: 5 ml, 0.5 mM; CTAB: 5 ml, 0.2 M) under vigorous stirring in 30 °C water bath. The growth process can be divided into three steps. First, the growth solution was prepared by mixing the HAuCl_4_ solution (25 ml, 1 mM), the silver nitrate (AgNO_3_) solution (1.8 ml, 4 mM) with the CTAB–sodium oleate (NaOL) solution (25 ml Milli-Q water; CTAB: 0.90 g; NaOL: 0.15 g) in 50 ml cuvette. Second, the hydrochloric acid (HCl) (150 μl, 37 wt. % in water) was added to tune the pH. Finally, the ascorbic acid (125 μl, 64 mM) was injected into the solution to reduce the Au(III) (yellow) into Au(I) (colorless) and finally the nanoseeds solution (200 μl) was added into the solution for growth. The cuvette was kept in 30 °C water bath overnight after gently shaken. The average LSPR peak of nanorods can be tuned by adjusting the amount of CTAB, NaOL, AgNO_3_ and HCl, accordingly.

All the nanorods used in this study were from the same cuvette to make sure they were in exactly the same chemical environment. The innate size variability provides nanorods with different aspect ratios. The nanoparticles were centrifuged and washed three times with Milli-Q water to remove the CTAB and NaOL. After being diluted for 100 times, the nanorod solution was dropped onto a quartz substrate with co-localization markers. To dry the sample, the substrate was put into a vacuum desiccator connected to a water circulation vacuum pump.

To correlate the SERS and PL spectrum of single nanorods with and without molecules, it is important to allow the molecule to adsorb only on the nanorod surface other than the quartz surface. Therefore, the surface of the quartz substrate was modified with special functional groups to prevent non-specific binding of the target fluorescence molecule. The modification was carried out by chemical vapor deposition of trichloro(1H, 1H, 2H, 2H-perfluorooctyl)silane (PFS) from Aldrich^49^. The quartz substrate was placed in a sealed vessel. A few drops of PFS was dispensed on the bottom of the vessel. The vessel was kept in an oven at 80 °C for 6 h to make sure the vapor of PFS fully reacted with the OH groups on the quartz surface. After the PL measurement, the quartz substrate was immersed in the solution containing the modified MGITC molecule for 1 h. Then, the sample was thoroughly rinsed by copious amount of Milli-Q water and immersed in ethanol for more than 4 h before the Raman measurement.

Silver nanoparticles were synthesized following the Lee–Meisel citrate reduction method^50^. After twice centrifuging and washing, an aliquot of silver nanoparticle solution was incubated with 1 ml of 1 mM NaCl+10^−7^ M Rhodamine 6G for 2 h (ref. 18). Afterwards, the sample was rinsed with water and finally blow-dried with nitrogen flow.

### Fabrication of markers on quartz substrate and SEM characterization

Microelectromechanical techniques were used to fabricate the markers on the quartz substrate. See Supplementary Fig. 1 for detail. We correlate the single-nanoparticle spectra and the morphology of the nanorods by using this substrate for co-localization in the dark-field microscope and a SEM (Hitachi S-4800)^51^. To obtain clear SEM images, 3 nm platinum was deposited after the PL and SERS measurement.

### Single-nanoparticle spectroscopy

Single-nanoparticle spectroscopic measurements were carried out on a transmission dark-field microscope (Leica, inverted) combined Renishaw inVia Raman instrument^51^. The dark-field white light illumination was achieved by a 100 W halogen lamp through an oil-immersion condenser (NA=1.2–1.44, Leica). The signal (elastic scattering, PL and SERS) was collected by a same objective (NA=0.75, 50 × , Leica). After passing through a 30 μm slit, the signal was dispersed on a 150 grooves per mm grating and finally collected by the CCD. The power of the 633 nm laser was set as low as 4.4 μW to measure the PL and SERS spectra and the spectra were acquired for 10 s with six accumulations, with no observable change between each acquisition. The polarization of both the laser and the white light were kept parallel to the longitudinal axis of the nanorods in corresponding measurement by adjusting a half-wave plate (PL and SERS measurements) or a high-transmission polarizer (scattering measurements). As shown in the Supplementary Fig. 11, although the absolute intensity of SERS and SERS background changes when laser polarization is changed relative to the long axis of the nanorod, the normalized SERS spectra show nearly identical feature at different polarization angles. The scattering spectra were measured before and after PL or SERS spectra acquisitions of every nanorod to confirm no morphological change happening during the laser illumination. All spectra have been calibrated to take the wavelength dependent response of the system into account^51^. The wavelength dependent response of the system was measured by using an AvaLight-HAL-CAL calibrated light source.

### TERS experiments and preparation of the gold(111) single crystal

Gold(111) single-crystal beads were prepared according to the Clavilier's method commonly used in surface science. The gold(111) single crystal was electrochemically polished to obtain a clean and atomically flat surface. After annealed with a butane flame, the single crystal was cooled down in the Ar atmosphere for the PL measurement. For TERS experiments, an atomic layer of palladium was deposited on the gold(111) surface, then the 4-chlorophenyl isocyanide molecule was adsorbed on the Pd/Au(111) surface to form a self-assembled monolayer as the Raman probe. TERS experiments were performed on a home-built set-up^52^ under 633 nm excitation at 0.5 mW with different Au tips but the same gold(111) single-crystal bead.

### Theoretical simulations

A commercial finite-element method simulation software (COMSOL multiphysics package 4.4) was used for theoretical simulations. Supplementary Fig. 4 shows a sketch of the simulated geometry. The size of nanorods were taken from the SEM images. Due to the 3 nm Pt deposition, the total length as well as the diameter of the nanorod were slightly adjusted to match the measured dark-field scattering spectra. See Supplementary Note 1 for detailed simulation parameters.

### Data availability

The data that support the findings of this study are available from the corresponding authors upon reasonable request.

## Additional information

**How to cite this article:** Lin, K.-Q. *et al*. Plasmonic photoluminescence for recovering native chemical information from surface-enhanced Raman scattering. *Nat. Commun.* **8,** 14891 doi: 10.1038/ncomms14891 (2017).

**Publisher's note:** Springer Nature remains neutral with regard to jurisdictional claims in published maps and institutional affiliations.

## Supplementary Material

Supplementary Information Supplementary Figures, Supplementary Notes, Supplementary Methods and Supplementary References

## Figures and Tables

**Figure 1:**
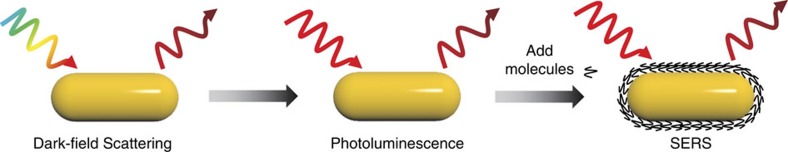
Schematic diagram for the quantitative experimental study. To correlate the LSPR, PL and SERS of single nanorods, the dark-field scattering and the PL signal are first measured in the absence of molecules, and SERS spectra were then measured after molecules adsorption.
