# Supplementary material for: Plasmonic photoluminescence for recovering native chemical information from surface-enhanced Raman scattering
Source: Nat Commun. 2017 Mar 28;8:14891. doi: 10.1038/ncomms14891 (PMC5379060; doi:10.1038/ncomms14891)
Supplement: Supplementary Information — Supplementary Figures, Supplementary Notes, Supplementary Methods and Supplementary References [file ncomms14891-s1.pdf]

Methods

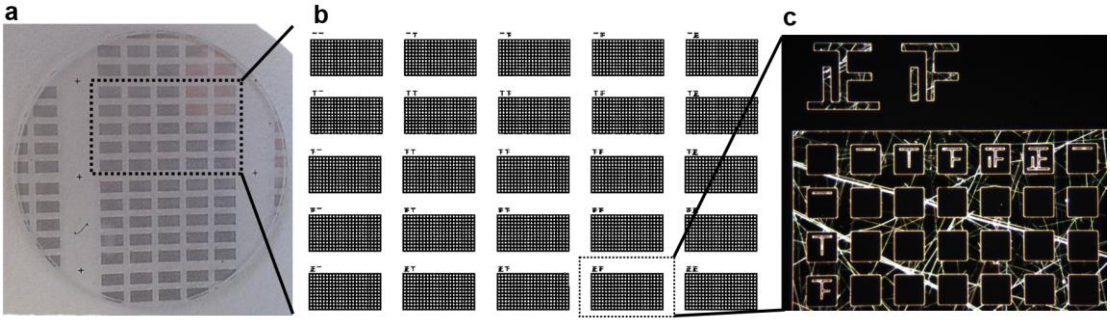

Supplementary Figure 1. Substrate used to localize and characterize individual plasmonic structures. (**a**) A photo showing the quartz substrate, which is divided into periods of 5×5 units as depicted in (**b**). Each unit is subdivided into 22×11 square regions of 60 μm^2, as illustrated by the SEM image in (**c**). The Chinese numerals allow locating each individual region, which made it possible to characterize the same plasmonic nanorods in different measurements.

Single nanoparticle spectroscopy

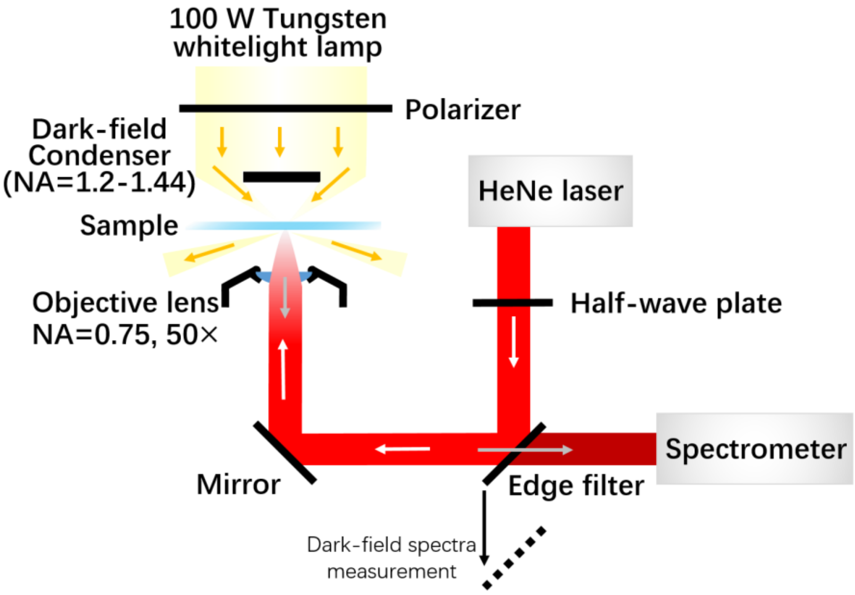

Supplementary Figure 2. Scheme of the set-up used for the optical characterization of the structures. The setup combined a homebuilt dark-field microscope with a Renishaw inVia Raman spectrometer. The sample was illuminated from the bottom by a 633 nm HeNe laser focused by an objective of 0.75 numerical aperture (50×, Leica), or from the top by a 100 W broadband halogen lamp focused by an oil-immersion dark field condenser (NA=1.2-1.44, Leica). The nanorods in the sample were located at the bottom interface of the quartz substrate, i.e. facing down. The signal was collected by the same objective (NA=0.75, 50x), and directed towards a spectrometer. A polarizer and a half-wave plate were used to polarize the broadband light and the laser, respectively, along the axis of the nanorods being characterized.

MGITC modification

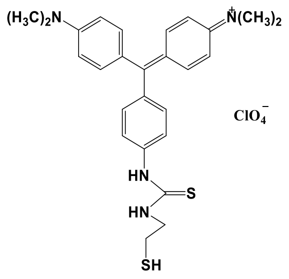

Supplementary Figure 3. The chemical structure of the modified MGITC molecule. The MGTIC (malachite green isocyanate) molecule was reacted with cysteamine to obtain modified MGITC containing a thiol group for a strong interaction with the Au nanorods.

Theoretical simulations

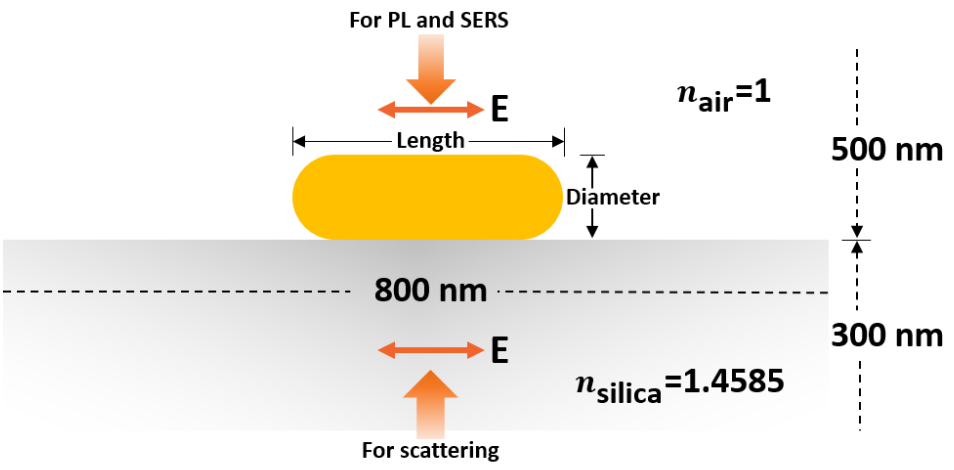

Supplementary Figure 4. Schematic of the numerical model. The gold nanorod was placed over a silica substrate. For the scattering simulations, the excitation light is incident from the bottom quartz substrate. To calculate the PL and SERS enhancement, the incident light comes from the top air part. The polarization of the light is always parallel to the nanorods axis.

Supplementary Note 1. Simulation method

The geometry to be solved is displayed in Supplementary Fig. 4. A rotationally-symmetric gold rod capped by semispheres is deposited over a quartz substrate characterized by optical index n_{silica}=1.4585. Except for the bottom contact region, the rod is surrounded by air n_{air} =1. The size parameters are based on the SEM image of the five nanorods in Fig. 3a-e in the main text. Because the quartz substrate is non-conductive, we sputtered about 3 nm Pt to obtain a good SEM image. Thus the exact size used for the simulations has been adjusted slightly to improve the agreement with the scattering spectra. The final diameter and length of the nanorods used for the simulations are 51 nm : 125.5 nm (Nanorod 1), 49 nm : 126.5 nm (Nanorod 2), 53nm : 137.5 nm (Nanorod 3), 42 nm : 127.5 nm (Nanorod 4), and 38nm : 122.5 nm (Nanorod 5). For SERS and PL studies, the excitation wavelength was 633 nm.

The permittivity of gold is taken from Johnson and Christy[1]. For direct comparison with experiments, illumination normal to the substrate either from the vacuum (for PL and SERS) or from the substrate (for scattering) is considered, and the polarization is always parallel to the rods. The excitation wavelength for PL and SERS was set to 633 nm. The simulations are solved using COMSOL, for a simulation volume that extends 500 nm over the substrate and 300 nm below it, and, laterally, comprises a square with 800 nm long sides and the rod at its center. Perfect matched layers, which are 400 nm away from the nanorod, are used at the boundaries. The mesh size over the whole nanorod are set to 2 nm and the mesh at the surface region are finer with mesh sizes down to 0.5 nm. As for the simulation regions outside the nanostructure, the maximal mesh size is defined as one-sixteenth of wavelength. To calculate the elastic scattering of the nanorods, a scattered-field formulation was used. The scattered fields, with electric component E_s and magnetic component H_s, were obtained by subtracting the total signal with the background field from an incident plane wave in the absence of the nanorod. This background was obtained analytically via the Fresnel reflection and transmission coefficients at the vacuum-substrate interface. The scattering power over the 4π solid angle was calculated by integrating the relative Poynting vector over an enveloping surface of the nanorod as

$$P_{\mathrm{sca}} = \frac{1}{2}\int Re(\mathbf{E}_{\mathrm{s}} \times \mathbf{H}_{\mathrm{s}}^{*}) \cdot \boldsymbol{e}_{\mathrm{r}} dS \quad (1)$$

Supplementary Note 2. Correlation of the PL radiation enhancement factor and scattering. For simple systems such as nanorods, it was found useful in the main manuscript to assume that the scattering and the integral over the local intensity present the same frequency dependence. We thus simulate the five gold nanorods to explore the relation between the integrated intensity enhancement $\int |\mathbf{E}_{\mathrm{ins}}(\omega,\mathbf{r})/E_0|^2 dv$ and the scattering, where $\mathbf{E}_{\mathrm{ins}}(\omega,\mathbf{r})/E_0$ is the local field enhancement factor inside the nanoparticle at position $\mathbf{r}$ excited at ω angular frequency. As shown in Supplementary Fig. 5a, the calculated scattering and $\int |\mathbf{E}_{\mathrm{ins}}(\omega,\mathbf{r})/E_0|^2 dv$ spectra indeed present an almost identical peak, which validates our assumption. Furthermore, the ratio of $\int |\mathbf{E}_{\mathrm{ins}}(\omega,\mathbf{r})/E_0|^2 dv$ to scattering (Supplementary Fig. 5b) varies slowly with ω and is almost the same for the five nanorods, independent of the LSPR resonant energy (see Supplementary Note 3 and 4 for further discussion).

We further derived in the main text that the PL enhancement at ω due to the plasmonic response can be determined by $\int |\mathbf{E}_{\mathrm{ins}}(633\,nm,\mathbf{r})/E_0|^2 \cdot |\mathbf{E}_{\mathrm{ins}}(\omega,r)/E_0|^2 dv$, which takes into account not only the enhancement at ω but also the strength of the local absorption at the incident 633 nm wavelength $\mathbf{E}_{\mathrm{ins}}(633\,nm,\mathbf{r})$. To obtain this equations, we used a simplified version of the reciprocity theorem[2, 3] [$\Gamma(\mathbf{r},\omega_{\mathrm{em}}) \propto \omega_{\mathrm{em}}^4 |\mathbf{E}_{\mathrm{in}}(\mathbf{r},\omega_{\mathrm{em}})/E_0|^2$, with Γ the decay rate of the excited dipoles or, similarly, currents]. We disregard differences of orientation between $\mathbf{E}_{\mathrm{in}}(\mathbf{r},\omega_{\mathrm{em}})$ and the induced dipoles at ω_{em}, and only consider that the total emission behaves as the light emitted backwards with same polarization as the incident light. These assumptions are expected to correctly approximate the emitted signal when the longitudinal dipolar mode of the nanorods determines the response, so that a single polarization is dominant and the radiation pattern remains dipolar. The ω_{em}^4 factor appears because of the frequency dependence of the emission from a dipole. More care would be necessary for systems where different plasmonic modes plays an important role, such as the case of two orthogonal plasmonic modes that has been recently discussed.[4] Supplementary Fig. 5c demonstrates that $\int |\mathbf{E}_{\mathrm{ins}}(\omega,\mathbf{r})/E_0|^2 dv$ (solid lines) and $\int |\mathbf{E}_{\mathrm{ins}}(633\,nm,\mathbf{r})/E_0|^2 \cdot |\mathbf{E}_{\mathrm{ins}}(\omega,\mathbf{r})/E_0|^2 dv$ (open dots) show an almost identical dependence with ω for the rods under consideration (both signals are normalized to their maximum). Thus, for a given nanorod, $\mathbf{E}_{\mathrm{ins}}(633\,nm,\mathbf{r})$ can be neglected when looking at the dependence with emission frequency.

Last, the $\int |\mathbf{E}_{\mathrm{ins}}(633\,nm,\mathbf{r})/E_0|^2 \cdot |\mathbf{E}_{\mathrm{ins}}(\omega,\mathbf{r})/E_0|^2 dv$ dependence was obtained by assuming that the PL is an incoherent process, in the sense that we do not consider the relative phases of $\mathbf{E}_{\mathrm{in}}(\mathbf{r},\omega_{exc})$ and $\mathbf{E}_{\mathrm{in}}(\mathbf{r},\omega_{em})$ but rather operate directly with the absolute values. A similar approach but assuming all processes are coherent (and with a fixed phase difference between them) leads to a PL signal that is proportional to $|\int(\mathbf{E}_{\mathrm{ins}}(633\,nm,\mathbf{r})/E_0) \cdot$

$(\mathbf{E}_{\text{ins}}(\omega,\mathbf{r})/E_0)dv|^2$. Supplementary Fig. 5d compares the results of both integrals. The result for the simple coherent process considered here shows almost identical spectrum to the incoherent case.

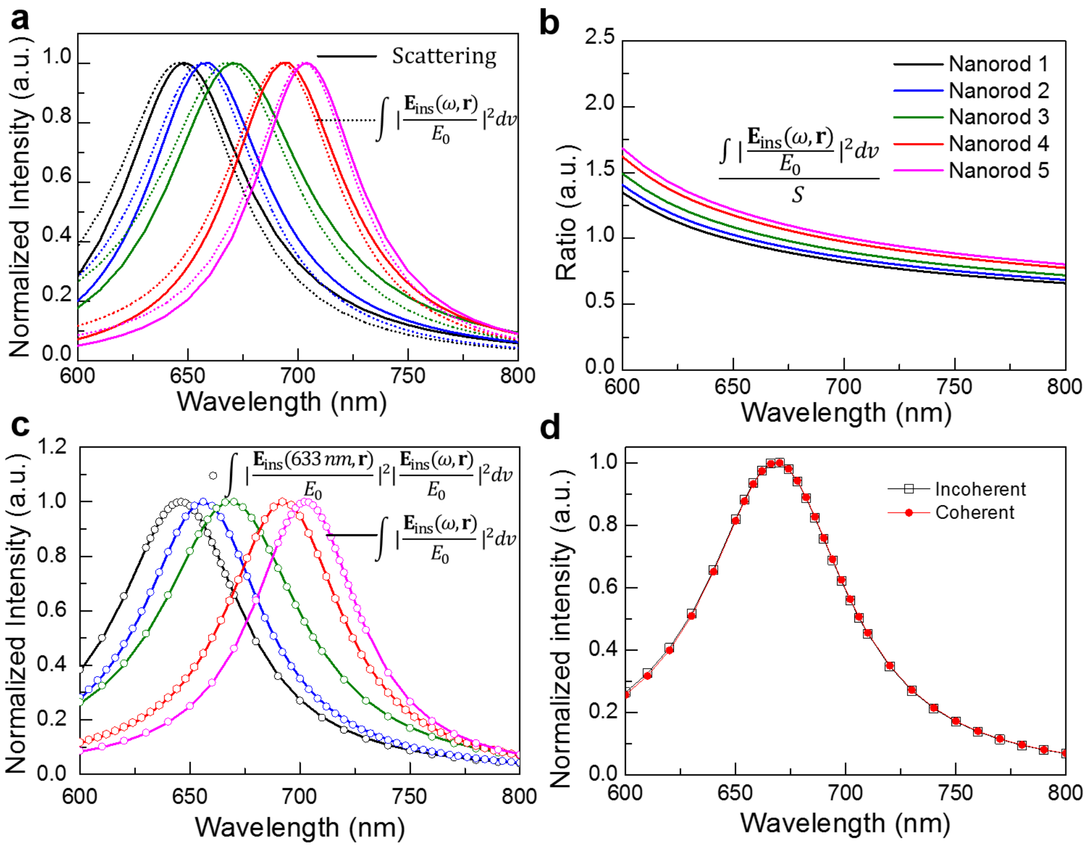

Supplementary Figure 5. Correlation of the PL radiation enhancement factor and scattering. (**a**) Normalized scattering spectra (solid lines) and integrated local field intensity enhancement factor inside the nanorods $\int|\mathbf{E}_{\text{ins}}(\omega,\mathbf{r})/E_0|^2dv$ (dots lines), obtained from the simulations of the five different gold nanorods on a quartz substrate. (**b**) Ratio of $\int|\mathbf{E}_{\text{ins}}(\omega,\mathbf{r})/E_0|^2dv$ to the elastic scattering of the five gold nanorods. (**c**) Integrated local field intensity enhancement factor $\int|\mathbf{E}_{\text{ins}}(\omega,\mathbf{r})/E_0|^2dv$ (solid lines) and integral over the local intensity at the incident (633 nm) and emission wavelengths $\int|\mathbf{E}_{\text{ins}}(633\,nm,\mathbf{r})/E_0|^2\cdot|\mathbf{E}_{\text{ins}}(\omega,\mathbf{r})/E_0|^2dv$ (open dots), for the five different nanorods. (**d**) PL electromagnetic enhancement factors, $\int|\mathbf{E}_{\text{ins}}(633\,nm,\mathbf{r})/E_0|^2\cdot|\mathbf{E}_{\text{ins}}(\omega,\mathbf{r})/E_0|^2dv$ (open black squares) and $|\int(\mathbf{E}_{\text{ins}}(633\,nm,\mathbf{r})/E_0)\cdot(\mathbf{E}_{\text{ins}}(\omega,\mathbf{r})/E_0)dv|^2$ (filled red circles), obtained with the assumption that the PL is an incoherent and coherent process, respectively. All results are calculated as a function of the emission wavelength and the integrals extend over the interior of the nanorods.

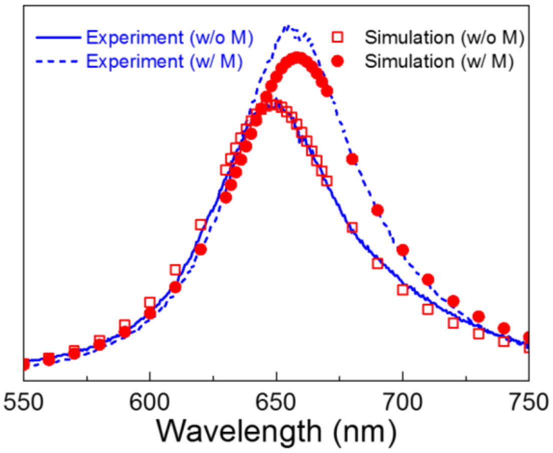

Supplementary Figure 6. LSPR shift induced by adsorbed molecules. Experimental scattering of Nanorod 1 of the main text before (solid line) and after (dash line) molecule adsorption, and simulated scattering for Nanorod 1 before (open square) and after (solid sphere) molecule adsorption. A 1.45 nm shell layer (n=1.4) was added to the nanorod surface to simulate a monolayer of the modified MGITC molecule. The simulated scattering spectrum of the nanorod after the molecule adsorption leads not only to a red shift in the peak position but also to an increase in intensity, which matches well with the experimental result.

Supplementary Note 3. Comparison of the SERS enhancement factor and PL enhancement factor

When the SERS signal is divided by the PL according to the model in the main text (Equations 1-4), it yields

$$\frac{SERS}{PL} \propto \frac{\sigma_{\mathrm{Raman}}(\omega_{\mathrm{exc}},\omega_{\mathrm{em}}) \int \left|\frac{\mathbf{E}_{\mathrm{out}}(\mathbf{r},\omega_{\mathrm{exc}})}{E_0}\right|^2 \left|\frac{\mathbf{E}_{\mathrm{out}}(\mathbf{r},\omega_{\mathrm{em}})}{E_0}\right|^2 \mathrm{d}V'}{\eta(\omega_{\mathrm{exc}},\omega_{\mathrm{em}})\,\omega_{\mathrm{em}}^4 \int \left|\frac{\mathbf{E}_{\mathrm{ins}}(\mathbf{r},\omega_{\mathrm{exc}})}{E_0}\right|^2 \left|\frac{\mathbf{E}_{\mathrm{ins}}(\mathbf{r},\omega_{\mathrm{em}})}{E_0}\right|^2 dV} \quad (2)$$

where $\mathbf{E}_{\mathrm{out}}(\mathbf{r},\omega_{\mathrm{exc}})/E_0$ and $\mathbf{E}_{\mathrm{out}}(\mathbf{r},\omega_{\mathrm{em}})/E_0$ are the local field enhancement factor at the excitation and emission energy, respectively, at the position $\mathbf{r}$. For the PL in the denominator, $\mathbf{r}$ refers to the position inside the rods, while the Raman signal in the numerator is integrated over the position of the molecules, i.e., a 1 nm thin layer covering the nanoparticle in these calculations. $\sigma_{\mathrm{Raman}}(\omega_{\mathrm{exc}},\omega_{\mathrm{em}})$ is the Raman cross sections, and $\eta(\omega_{\mathrm{exc}},\omega_{\mathrm{em}})$ is related to the bulk PL signal, for the same excitation and emission frequencies. The derivation of the SERS expression used reciprocity to relate the emission from the molecules to the local near fields, under similar assumptions as previously discussed for the PL. We have also assumed that we are treating off-resonant Raman or that –in the case of resonant Raman- the coupling strengths between the plasmon and the electronic level is not large enough to reach the strong coupling regime. Last, we neglect quantum effects that may emerge in state-of-the-art configurations as we push plasmonics to its limits.[5,6] If we look only at the dependence with the electromagnetic response, we obtain

$$\frac{SERS}{PL} \propto \frac{\int \left|\frac{\mathbf{E}_{\mathrm{out}}(\mathbf{r},\omega_{\mathrm{exc}})}{E_0}\right|^2 \left|\frac{\mathbf{E}_{\mathrm{out}}(\mathbf{r},\omega_{\mathrm{em}})}{E_0}\right|^2 \mathrm{d}V'}{\int \left|\frac{\mathbf{E}_{\mathrm{ins}}(\mathbf{r},\omega_{\mathrm{exc}})}{E_0}\right|^2 \left|\frac{\mathbf{E}_{\mathrm{ins}}(\mathbf{r},\omega_{\mathrm{em}})}{E_0}\right|^2 dV} \quad (3)$$

which is plotted in Supplementary Fig. 7a for the five simulated rods. The ratio vary slowly with emission wavelength and only depend weakly on the position of the LSPR. For a perfectly constant ratio, and the bulk PL_{bulk} proportional to $\omega_{\mathrm{em}}^4\eta(\omega_{\mathrm{exc}},\omega_{\mathrm{em}})$, we have $\sigma_{\mathrm{Raman}} \propto SERS/PL \times PL_{\mathrm{bulk}}$, which is the magnitude plotted in Fig. 4d in the main text.

To further analyze the behavior of the ratio in Supplementary Equation 3, the calculated SERS electromagnetic enhancement factor $\int |\mathbf{E}_{\mathrm{out}}(\mathbf{r},\omega_{\mathrm{exc}})/E_0|^2 |\mathbf{E}_{\mathrm{out}}(\mathbf{r},\omega_{\mathrm{em}})/E_0|^2 \mathrm{d}V'$, the PL enhancement factor $\int |\mathbf{E}_{\mathrm{ins}}(\mathbf{r},\omega_{\mathrm{exc}})/E_0|^2 |\mathbf{E}_{\mathrm{ins}}(\mathbf{r},\omega_{\mathrm{em}})/E_0|^2 \mathrm{d}V$ and the elastic scattering of the five nanorods are shown in Supplementary Fig. 7b, all

normalized to their corresponding maximum. As expected, all three magnitudes depends on ω_{em} on a similar fashion, with a clear peak indicating the enhancement due to the LSPR. The scattering and its similarity with the PL integral becomes important for the simplified Equations 2-5 in the main text. Discussion on the different approximations involved in the normalization of the SERS signal is included in the next Note.

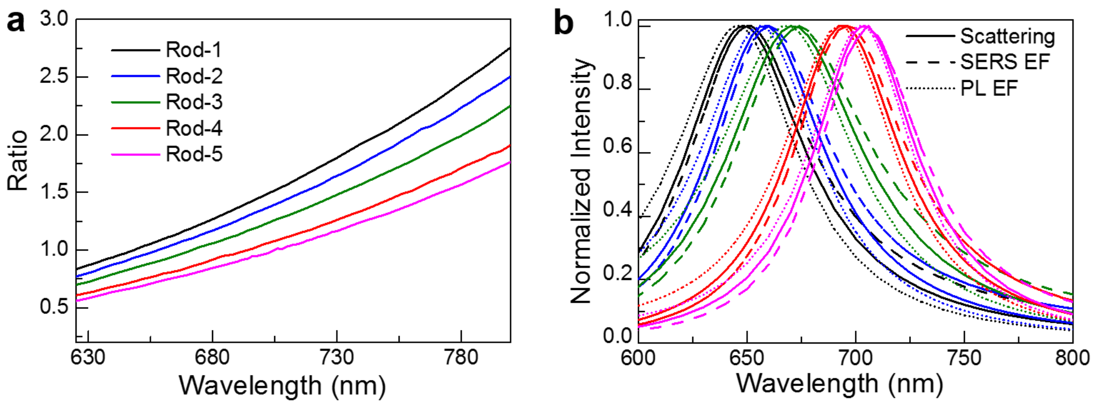

Supplementary Figure 7. Comparison of the enhancement factor of SERS and PL. (**a**) The calculated ratio of the SERS enhancement factor $\int |\mathbf{E}_{\text{out}}(\mathbf{r},\omega_{exc})/E_0|^2 |\mathbf{E}_{\text{out}}(\mathbf{r},\omega_{\text{em}})/E_0|^2 \text{dV}'$ to the PL enhancement factor $\int |\mathbf{E}_{\text{ins}}(\mathbf{r},\omega_{\text{exc}})/E_0|^2 |\mathbf{E}_{\text{ins}}(\mathbf{r},\omega_{\text{em}})/E_0|^2 \text{dV}$ of the five nanorods (Supplementary Equation 3), in arbitrary units. (**b**) The elastic scattering (solid lines), and the SERS $\int |\mathbf{E}_{\text{out}}(\mathbf{r},\omega_{exc})/E_0|^2 |\mathbf{E}_{\textbf{out}}(\mathbf{r},\omega_{\text{em}})/E_0|^2 \text{dV}'$ (dashed lines) and PL $\int |\mathbf{E}_{\text{ins}}(\mathbf{r},\omega_{\text{exc}})/E_0|^2 |\mathbf{E}_{\text{ins}}(\mathbf{r},\omega_{\text{em}})/E_0|^2 \text{dV}$ (dotted lines) electromagnetic enhancement factors, for the five nanorods simulated.

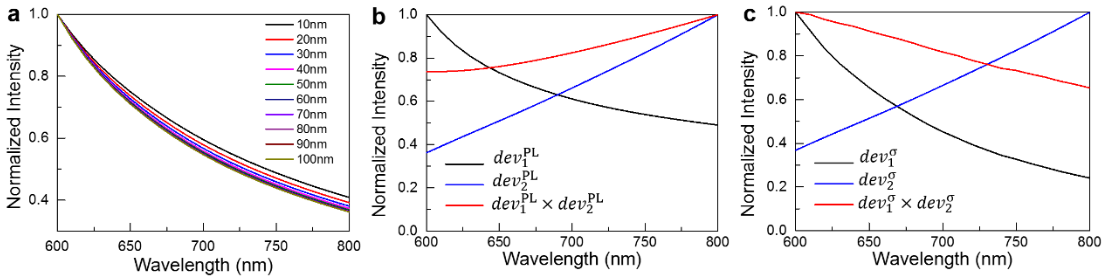

Supplementary Figure 8. Deviation evaluation. (**a**) The wavelength dependency of the near field inside a 2 μm thick gold flat film as a function of the integrating thickness. (**b**) Calculated $dev_1^{\text{PL}} = \int |\mathbf{E}_{\text{ins}}(\mathbf{r},\omega_{\text{em}})/E_0|^2 |\mathbf{E}_{\text{ins}}(\mathbf{r},\omega_{\text{em}})/E_0|^2 \text{dV}/S(\omega_{\text{em}})$ (black, similar to the black line in Supplementary Fig. 5b), calculated $dev_2^{\text{PL}} = 1/\int |\mathbf{E}_{\text{flat}}^{\text{ins}}(\mathbf{r},\omega_{exc})/E_0|^2 |\mathbf{E}_{\text{flat}}^{\text{ins}}(\mathbf{r},\omega_{\text{em}})/E_0|^2 \text{dV}$ (blue) and the total deviation $dev_1^{\text{PL}} \times dev_2^{\text{PL}}$(red). (**c**) Calculated $dev_1^{\sigma} = \int \left|\frac{\mathbf{E}_{\text{ins}}(\mathbf{r},\omega_{\text{em}})}{E_0}\right|^2 \left|\frac{\mathbf{E}_{\text{ins}}(\mathbf{r},\omega_{\text{em}})}{E_0}\right|^2 \text{dV}/\int \left|\frac{\mathbf{E}_{\text{out}}(\mathbf{r},\omega_{\text{em}})}{E_0}\right|^2 \left|\frac{\mathbf{E}_{\text{out}}(\mathbf{r},\omega_{\text{em}})}{E_0}\right|^2 \text{dV}'$ (black line, reciprocal of the black line in Supplementary Fig. 7a), calculated $dev_2^{\sigma} = 1/\int |\mathbf{E}_{\text{flat}}^{\text{ins}}(\mathbf{r},\omega_{\text{exc}})/E_0|^2 |\mathbf{E}_{\text{flat}}^{\text{ins}}(\mathbf{r},\omega_{\text{em}})/E_0|^2 \text{dV}$(blue line) and the total deviation $dev_1^{\sigma} \times dev_2^{\sigma}$ (red). All calculations in **b**, **c** were performed for nanorod-1.

Supplementary Note 4. A closer look at the assumptions

In the main text, we occasionally assumed that integrals on the square of the fields were proportional to the elastic scattering S, an approximation useful for simple systems. Nonetheless, due to the inevitable losses of the material, this proportionality does not strictly hold and the integrated square of the near field outside the nanorod is not strictly

proportional to that inside the nanorod, as can be seen from Supplementary Fig. 5a and Supplementary Fig. 7b, respectively (notice that the integral plotted in the latter figure also consider the intensity of the fields at the excitation frequency). In a relatively wide wavelength range like 600 – 800 nm, the corresponding ratios vary smoothly with ω_{em} but are actually not flat, which, to some extent, could affect the experimental results.

On the other hand, $PL_{\mathrm{bulk}}(\omega_{\mathrm{exc}}, \omega_{\mathrm{em}})$ is not just proportional to $\omega_{\mathrm{em}}^4 \eta(\omega_{\mathrm{exc}}, \omega_{\mathrm{em}})$, since the $PL_{\mathrm{bulk}}(\omega_{\mathrm{exc}}, \omega_{\mathrm{em}})$ signal measured for the flat surface is affected by the wavelength-dependent field response inside the flat metal surface. More exactly, by applying Equation 1 of the main text

$$PL_{\mathrm{bulk}}(\omega_{\mathrm{exc}}, \omega_{\mathrm{em}}) \propto \mathrm{I}_0 \omega_{\mathrm{em}}^4 \eta(\omega_{\mathrm{exc}}, \omega_{\mathrm{em}}) \int \left|\frac{\mathbf{E}_{\mathrm{flat}}^{\mathrm{ins}}(\mathbf{r},\omega_{\mathrm{exc}})}{E_0}\right|^2 \left|\frac{\mathbf{E}_{\mathrm{flat}}^{\mathrm{ins}}(\mathbf{r},\omega_{\mathrm{em}})}{E_0}\right|^2 \mathrm{dV} \quad (4)$$

where $\mathbf{E}_{\mathrm{flat}}^{\mathrm{ins}}(\mathbf{r}, \omega_{\mathrm{exc}})$ is the electric field on the volume V inside the flat gold surface. In the main text, the integral is assumed to be almost independent of emission frequency. However, the calculation of the wavelength dependency of the near field $\int |\mathbf{E}_{\mathrm{flat}}^{\mathrm{ins}}(\mathbf{r}, \omega_{\mathrm{exc}})/E_0|^2 |\mathbf{E}_{\mathrm{flat}}^{\mathrm{ins}}(\mathbf{r}, \omega_{\mathrm{em}})/E_0|^2 \mathrm{dV}$ inside a 2 μm thick gold flat film as a function of the integrating thickness (Supplementary Fig. 8a), indicates that this assumption is not exactly valid when considering a wide wavelength range: the integral again depends on ω_{em} in a well-behaved manner.

A further approximation was ignoring the influence on the integrals of the plasmonic enhancement at the rod excitation frequency, which is shown not to introduce any significant deviation in Supplementary Fig. 5c.
We discuss in the following how these effects affect the normalization of the PL by the scattering and of the Raman peaks by the background (Fig. 2c and Fig. 4d in the main text). Favorably for our purposes, we will see how the different errors introduced partially cancel.

In Supplementary Note 2, we have discussed the ratio $\int |\mathbf{E}_{\mathrm{ins}}/E_0|^2 dv/S$, which, when frequency independent, allows to relate the experimental results to the scattering. More strictly, to go from Equation 1 in the main text and Supplementary Equation 4 to $PL(\omega_{\mathrm{em}})/S(\omega_{\mathrm{em}}) \propto PL_{\mathrm{bulk}}(\omega_{\mathrm{em}})$,

$$dev_1^{\mathrm{PL}} \times dev_2^{\mathrm{PL}} = \frac{\int \left|\frac{\mathbf{E}_{\mathrm{ins}}(\mathbf{r},\omega_{\mathrm{exc}})}{E_0}\right|^2 \left|\frac{\mathbf{E}_{\mathrm{ins}}(\mathbf{r},\omega_{\mathrm{em}})}{E_0}\right|^2 \mathrm{dV}}{S(\omega_{\mathrm{em}})} \times \frac{1}{\int \left|\frac{\mathbf{E}_{\mathrm{flat}}^{\mathrm{ins}}(\mathbf{r},\omega_{\mathrm{exc}})}{E_0}\right|^2 \left|\frac{\mathbf{E}_{\mathrm{flat}}^{\mathrm{ins}}(\mathbf{r},\omega_{\mathrm{em}})}{E_0}\right|^2 \mathrm{dV}} \quad (5)$$

should not depend on ω_{em}. As observed in Supplementary Fig. 8b , the results from Supplementary Equation 5 depend on the emission frequency more weakly than either of the two individual factors in the expression, or than the simplified $\int |\mathbf{E}_{\mathrm{in}}/E_0|^2 dv/S$ (Supplementary Fig. 5b), which further justifies why the normalized spectra $PL(\omega_{\mathrm{em}})/S(\omega_{\mathrm{em}})$ in Fig. 1c in the main text recovers $PL_{\mathrm{bulk}}(\omega_{\mathrm{em}})$ for the flat surface.

On a similar manner, we can obtain from Supplementary Equation 2

$$\sigma_{\mathrm{Raman}}(\omega_{\mathrm{em}}) \propto \frac{SERS(\omega_{\mathrm{em}}) PL_{\mathrm{bulk}}(\omega_{\mathrm{exc}},\omega_{\mathrm{em}})}{Bg} \frac{\int \left|\frac{\mathbf{E}_{\mathrm{ins}}(\mathbf{r},\omega_{\mathrm{exc}})}{E_0}\right|^2 \left|\frac{\mathbf{E}_{\mathrm{ins}}(\mathbf{r},\omega_{\mathrm{em}})}{E_0}\right|^2 \mathrm{dV}}{\int \left|\frac{\mathbf{E}_{\mathrm{out}}(\mathbf{r},\omega_{\mathrm{exc}})}{E_0}\right|^2 \left|\frac{\mathbf{E}_{\mathrm{out}}(\mathbf{r},\omega_{\mathrm{em}})}{E_0}\right|^2 dV'} \frac{1}{\int \left|\frac{\mathbf{E}_{\mathrm{flat}}^{\mathrm{ins}}(\mathbf{r},\omega_{\mathrm{exc}})}{E_0}\right|^2 \left|\frac{\mathbf{E}_{\mathrm{flat}}^{\mathrm{ins}}(\mathbf{r},\omega_{\mathrm{em}})}{E_0}\right|^2 \mathrm{dV}} \quad (6)$$

where $\sigma_{Raman}(\omega_{em})$ is the Raman cross-section for no electromagnetic enhancement and the SERS signal would correspond in the experiments to the signal after subtracting the Raman background. Comparing Equation 7 in the main text and Supplementary Equation 6, we can define $\frac{\int \left|\frac{\mathbf{E}_{\mathrm{ins}}(\mathbf{r},\omega_{\mathrm{exc}})}{E_0}\right|^2 \left|\frac{\mathbf{E}_{\mathrm{ins}}(\mathbf{r},\omega_{\mathrm{em}})}{E_0}\right|^2 \mathrm{dV}}{\int \left|\frac{\mathbf{E}_{\mathrm{out}}(\mathbf{r},\omega_{\mathrm{exc}})}{E_0}\right|^2 \left|\frac{\mathbf{E}_{\mathrm{out}}(\mathbf{r},\omega_{\mathrm{em}})}{E_0}\right|^2 dV'}$ as deviation dev_1^{σ} and $\frac{1}{\int \left|\frac{\mathbf{E}_{\mathrm{flat}}^{\mathrm{ins}}(\mathbf{r},\omega_{\mathrm{exc}})}{E_0}\right|^2 \left|\frac{\mathbf{E}_{\mathrm{flat}}^{\mathrm{ins}}(\mathbf{r},\omega_{\mathrm{em}})}{E_0}\right|^2 \mathrm{dV}}$ as dev_2^{σ}. For $SERS(\omega_{\mathrm{em}}) \times PL_{\mathrm{bulk}}(\omega_{\mathrm{exc}}, \omega_{\mathrm{em}})/Bg$ (Fig. 4d in the manuscript) to be proportional to $\sigma_{\mathrm{Raman}}(\omega_{\mathrm{em}})$, the total deviation $dev_1^{\sigma} \times dev_2^{\sigma}$ should remain independent of the emission frequency. The simulation results in Supplementary Fig. 8c show that the two deviations resulting from the two assumptions are in the opposite direction and thus partially cancel. The total deviation is relatively small even for a relatively wide wavelength range, as desired to correctly determine $\sigma_{\mathrm{Raman}}(\omega_{\mathrm{em}})$.

Last, in Fig. 4 in the main text we compared the corrected SERS for the plasmonic rods to the Raman signal measured for the molecules adsorbed on a flat gold single crystal surface. For the latter, we did not take into account how the electromagnetic response of the flat surface affects the Raman spectrum of the molecules, because the

electric field at the surface varies relatively slowly with energy in the range studied. To take this effect into account, we obtain the corrected $\sigma_{\mathrm{Raman}}(\omega_{\mathrm{em}})$ (red line in Supplementary Fig. 9) for the molecules over the flat surface by dividing the measured Raman spectrum (black line in Supplementary Fig. 9) by the electromagnetic enhancement $\int |\frac{\mathbf{E}_{\mathrm{flat}}^{\mathrm{out}}(\mathbf{r},\omega_{\mathrm{exc}})}{E_0}|^2 |\frac{\mathbf{E}_{\mathrm{flat}}^{\mathrm{out}}(\mathbf{r},\omega_{\mathrm{em}})}{E_0}|^2 \mathrm{d}V'$. $\mathbf{E}_{\mathrm{flat}}^{\mathrm{out}}(\mathbf{r},\omega_{\mathrm{exc}})$ corresponds to the fields in the volume V' occupied by a 1.45 nm thick monolayer of molecules.

The new correction does not significantly modify our previous discussion, with the correction only weakly affecting the overall shape of the figure. Thus, the new corrected Raman spectrum of the molecules over a flat surface remains similar to the $SERS(\omega_{\mathrm{em}})PL_{\mathrm{bulk}}(\omega_{\mathrm{exc}},\omega_{\mathrm{em}})/Bg$ corrected values for the different rods. We notice that an exact correction may need to consider aspects such as dark field illumination or the orientation of the molecules, so that Supplementary Fig. 9 is mostly given as an illustration on how the influence of the electromagnetic enhancement on the relative strength of the peaks is small for the flat substrate. Additional work considering the subtle differences between these and other correction schemes and the influence of effects such as molecule orientation may help to further improve the agreement on the measurement of $\sigma_{\mathrm{Raman}}(\omega_{\mathrm{em}})$ for any substrate, but the results in Fig. 4 in the main text is highly satisfactory.

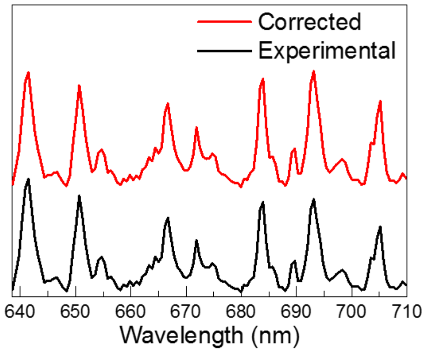

Supplementary Figure 9. Comparison of the experimental and deviation corrected Raman spectrum. The experimentally measured Raman spectrum of the MGITC on a gold(111) single crystal surface is shown as the black line and same spectra after correcting for the wavelength dependent electromagnetic response of the surface is shown as the red line. For convenience, the spectra were normalized and given against the absolute wavelength with 633 nm excitation.

Supplementary Note 5. Data processing method and comparison of the fitted background and PL

In Fig. 4 on the main text, we divided the measured Raman spectra by its background. The background was first fitted with the adaptive iteratively reweighted penalized least squares (AirPLS) method introduced by Eilers[7], which is commonly used in spectral analysis including Raman, surface-enhanced infrared absorption (SEIRA) and NMR spectroscopy. Using this method, the SERS spectra were split into two parts: the SERS peaks and SERS background. Then, each background-subtracted SERS spectrum was divided by its background spectrum, resulting in the corrected SERS spectrum.

The fitted background and PL spectra of five nanorods are shown in Supplementary Fig. 10 for comparison. The PL has been corrected according to Equation 3 in the main text to take into account the presence of the molecules. There is only a moderate intensity difference between the fitted background and the corrected PL spectra.

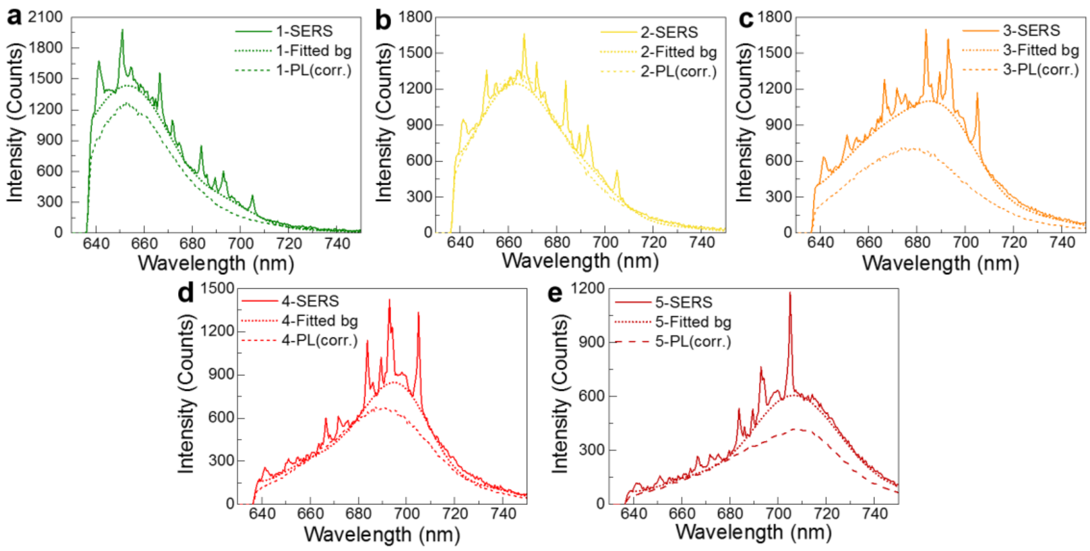

Supplementary Figure 10. Comparison of the fitted background and PL. (**a**)-(**e**) SERS spectra measured (solid line), fitted backgrounds (dotted line), and measured PL spectra after correction to incorporate the effect of the molecules (dashed line), for the five nanorods in Fig. 3 of the main text.

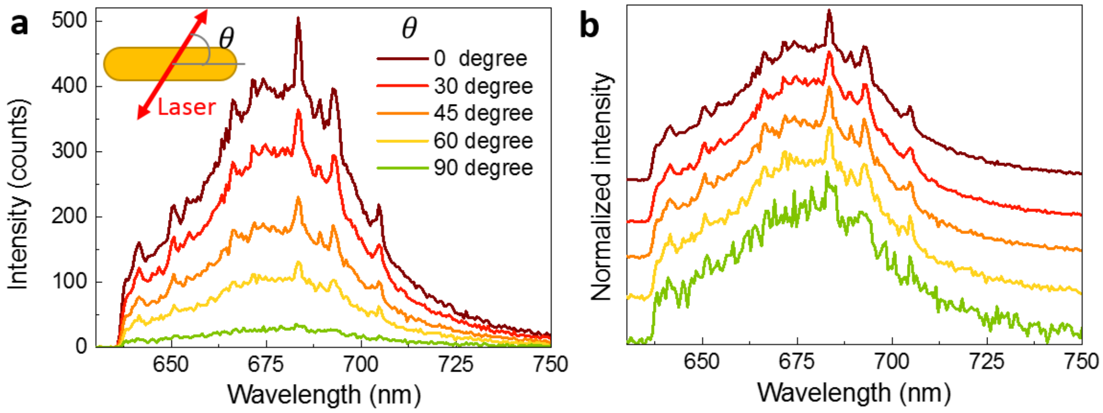

Supplementary Figure 11. Polarization dependent SERS spectra. (**a**) Laser polarization dependent SERS spectra of single gold nanorod. (**b**) Normalized laser polarization dependent SERS spectra in **a**. The different spectra are shifted vertically for clarity. Changing the laser polarization strongly affects the strength of the Raman lines and background but not the relative intensity of the Raman lines.

Supplementary References

1. Johnson, P. B. & Christy, R. W. Optical Constants of the Noble Metals. *Phys. Rev. B* **6**, 4370-4379 (1972).
2. Carminati, R., Nieto-Vesperinas, M. & Greffet, J.-J. Reciprocity of Evanescent Electromagnetic Waves. *J. Opt. Soc. Am. A* **15**, 706-712 (1998).
3. Bharadwaj, P., Deutsch, B. & Novotny, L. Optical Antennas. *Adv. Opt. Photon.* **1**, 438-483 (2009).
4. Andersen, S. K. H., Pors, A. & Bozhevolnyi, S. I. Gold Photoluminescence Wavelength and Polarization Engineering. *ACS Photonics* **2**, 432-438 (2015).

5. Roelli, Philippe, Christophe Galland, Nicolas Piro, and Tobias J. Kippenberg. Molecular Cavity Optomechanics as a Theory of Plasmon-enhanced Raman Scattering. *Nat. Nanotech.* 11, 164-169 (2015).
6. Schmidt, Mikolaj K., Ruben Esteban, Alejandro González-Tudela, Geza Giedke, and Javier Aizpurua. Quantum Mechanical Description of Raman Scattering from Molecules in Plasmonic Cavities. *ACS nano* 2016, 10, 6291-6298.
7. Eilers, P. H. A perfect smoother. *Anal. Chem.* **75**, 3631-3636 (2003).
